# Remote Management of Patients With Diabetic Macular Edema Receiving Long-Term Intravitreal Anti–Vascular Endothelial Growth Factor Therapy (RE-SHINE Study): Protocol for a Real-World Study

**DOI:** 10.2196/85764

**Published:** 2026-04-06

**Authors:** Shu Li, Yupeng Xu, Yan Xu, Xun Xu

**Affiliations:** 1 Shanghai General Hospital, Shanghai Jiao Tong University School of Medicine, Shanghai, China; 2 National Clinical Research Center for Eye Diseases, Shanghai, China; 3 Department of Ophthalmology, Shanghai General Hospital, Shanghai Jiao Tong University School of Medicine, No. 85 Wujin Road, Hongkou District, Shanghai, China, 86 63240090

**Keywords:** diabetic macular edema, remote management, anti-VEGF therapy, Behavior Change Wheel, treatment adherence, digital health, anti–vascular endothelial growth factor therapy

## Abstract

**Background:**

Long-term intravitreal anti–vascular endothelial growth factor therapy is the gold standard for treating diabetic macular edema (DME). However, poor treatment adherence in real-world settings often leads to suboptimal visual outcomes. Evidence-based remote management strategies are required to bridge the gap between clinical protocols and patient self-management.

**Objective:**

This study aims to describe a remote management program based on the Behavior Change Wheel model designed to increase treatment adherence and improve disease outcomes for patients with DME receiving long-term anti–vascular endothelial growth factor therapy, and to report the study protocol.

**Methods:**

The RE-SHINE study was structured into three phases: theoretical modeling, digital platform development, and a validation cohort study. The intervention included a smartphone-based app providing personalized health education, self-monitoring of vision (Amsler grid), injection reminders, and direct communication with health care providers. The efficacy of the remote management model was tested in a real-world cohort study. Participants were assessed at baseline and at each follow-up visit over 12 months. The primary outcome measure was best-corrected visual acuity, and secondary outcome measures included central retinal thickness and treatment adherence (injection rate and follow-up frequency).

**Results:**

A total of 1006 patients with DME underwent initial screening, and 958 (95.2%) patients met the inclusion criteria, with only 7 patients declining follow-up. We anticipate that the remote management system will be both feasible and acceptable for patients with DME. It is hypothesized that the intervention group would demonstrate significantly higher levels of treatment adherence and better visual preservation compared to the retrospective control group. In addition, we intend to assess the impact of the digital intervention on patient-reported outcomes, encompassing quality of life and self-efficacy. Participant recruitment began in September 2023 and was completed by the time of submission. The data analysis is yet to begin. The results are expected to be published in 2026.

**Conclusions:**

A Behavior Change Wheel–based remote management approach could be a scalable and sustainable method to enhance adherence in patients requiring long-term intravitreal therapy. If proven effective, this model could be integrated into routine ophthalmic care to mitigate the risk of vision loss due to undertreatment in DME.

## Introduction

Diabetic macular edema (DME) is a manifestation of diabetic retinopathy that impairs central vision and has a profoundly detrimental impact on vision-specific function, mobility, independence, and quality of life [1]. Over the past 15 years, intravitreal anti–vascular endothelial growth factor (VEGF) therapy injections have become the first-line therapy for patients with DME [2]. In clinical trials, patients received an average of 6 injections per year, resulting in a visual acuity improvement of 12 letters [3]. In contrast, in clinical practice, patients in China received an average of 2.9 injections during the initial year of treatment, resulting in a visual acuity improvement of 1.1 letters, a figure that was notably lower than the average of 5 letters in other countries [4]. These poor outcomes were attributed to the failure to administer timely and prescribed intravitreal anti-VEGF injections and to adhere to scheduled follow-up appointments [5].

A paucity of research has been conducted on intravitreal anti-VEGF injections, and there has been a lack of theoretical guidance and in-depth investigation into influencing factors [2,5-7]. Consequently, we conducted a study to investigate the factors influencing the timely intravitreal anti-VEGF therapy in Chinese patients [8]. The findings of this study identified that untimely intravitreal anti-VEGF injection was driven by psychological capability (eg, lack of relevant knowledge), social opportunity (eg, effective doctor-patient communication), physical opportunity (eg, high treatment costs), reflective motivation (eg, lack of confidence in the efficacy, unmet expectations), and automatic motivation (eg, fear of injections and fear of blindness). These specific determinants provided the direct rationale for the self-monitoring and education modules in the new platform. The “psychological capability (lack of relevant knowledge)” drives the design of the “structured education” module. The design of the “online Q&A” module is informed by the “social opportunity (effective doctor-patient communication)” initiative. The “reflective motivation (lack of confidence in efficacy, unmet expectations)” informs the design of the “treatment management and feedback” module. The “automatic motivation (fear of injections, fear of blindness)” is a key factor in the design of the “case sharing” module.

Concurrently, clinicians’ emphasis on standardized diagnosis and regimented treatment, with minimal consideration given to patient adherence, has not improved clinical outcomes or reduced the use of health care resources. Consequently, a comprehensive set of measures must be implemented to optimize patient management. Nevertheless, there is a paucity of domestic and international studies on the management of patients with DME. While digital management has been explored in chronic eye diseases such as glaucoma with promising outcomes [9-11] and demonstrating improved cost-effectiveness [12,13], DME presents unique challenges due to its complex injection schedules [14]. Digital health care is currently focused primarily on screening rather than patient management [15-18], and there is no evidence-based remote management framework specifically designed for this patient population.

The objective of this study is to develop a remote management system integrating the Behavior Change Wheel (BCW) framework with the technical features of digital platforms for patients with DME receiving long-term intravitreal anti-VEGF therapy.

## Methods

### Ethical Considerations

Ethical approval for the study protocol was obtained from the ethics committees of Shanghai General Hospital, Shanghai Jiao Tong University School of Medicine (2022-KY-021). In addition, this study was previously registered (ChiCTR2400090348). All participants were required to provide written informed consent, and their confidentiality was safeguarded through the implementation of anonymized data coding. The study entailed minimal risk and did not involve experimental treatments or invasive procedures. Participants could withdraw from the study at any time without consequence for their medical care.

### Design

**Figure 1.**
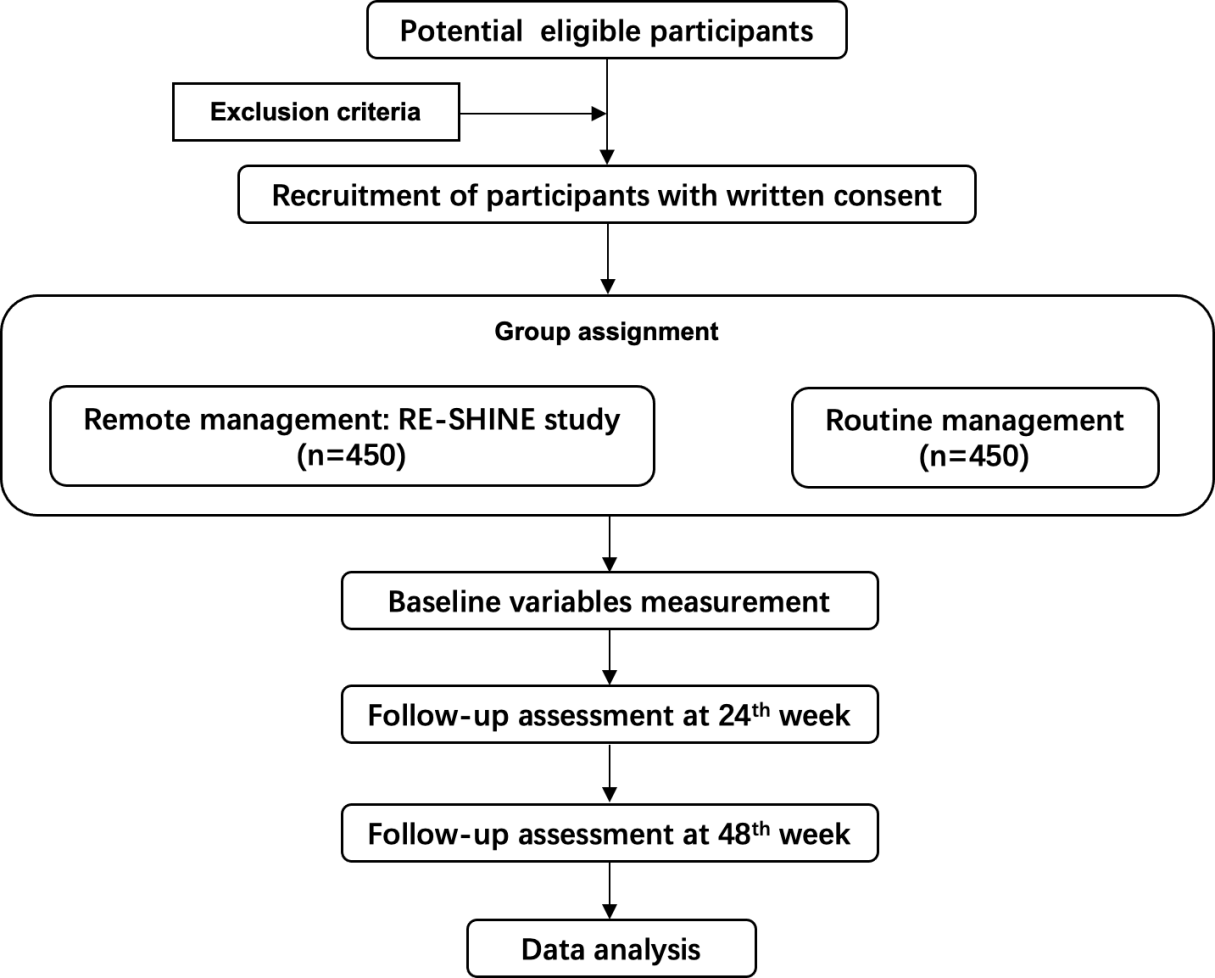
Study flowchart.

In this study, researchers conducted a three-stage observational study over 48 weeks, outlined as follows:

1. Theoretical modeling: A management system based on the BCW theory was constructed. The BCW was first proposed by Michie et al [19] as part of the development of the capability, opportunity, motivation–behavior (COM-B) theoretical model into the BCW theoretical framework. This theory has been formulated following a comprehensive synthesis of 19 related behavior change frameworks to support intervention designers in analyzing problems from a behavioral perspective, integrating interventions across three dimensions—capacity, opportunity, and motivation—to systematically select optimal intervention functions.
2. Platform development: Artificial intelligence questions and answers (Q&A), self-monitoring, and other modules were integrated.
3. A 48-week cohort study (remote group vs routine group) assessed the digital remote management model’s effects on visual acuity, retinal thickness, and injection rate. Figure 1 shows the study flow.

### Participation Recruitment and Eligibility Assessment

A convenience sampling methodology was used to recruit participants from 26 cities in 17 provinces between September 2023 and September 2025. The inclusion criteria were as follows: individuals with DME in one or both eyes who are older than 18 years, initiation of intravitreal anti-VEGF injections in the treated eye, central retinal thickness (CRT) of 300 µm or greater in the treated eye, best-corrected visual acuity (BCVA) in the treated eye at 39 to 73 letters using the Early Treatment Diabetic Retinopathy Study (ETDRS) chart 4-meter test (Snellen visual acuity ~20/40 to ~20/160), possession of a smartphone, and ability to use the study app (independently or with assistance from a regular caregiver). Exclusion criteria included previous surgical, laser, or intravitreal hormone therapy for retinal disease in the treated eye; confirmed Alzheimer disease, intellectual disability, severe psychiatric, or cognitive impairments that prevent the independent use of smartphone apps or the provision of informed consent; pregnancy; or absence of local follow-up.

Once suitable patients were identified, the researcher contacted them by telephone to provide an overview of the study, including its purpose, the methodology, data protection, and confidentiality. If suitable participants consented, they were requested to sign an informed consent form, thereby confirming their participation in the study.

### Sample Size

For conservative estimation, we consulted a large-scale study of patients with wet age-related macular degeneration (wAMD) [20] and used the ETDRS visual acuity scale as the primary end point (with *δ*=4.0 and s=16.2) and applied a 2-sided test (with 1 – β = .9 and *α*=.05); the missed visit rate was set at 20%. Using PASS 2021 software (NCSS, LLC) for estimation, the required minimum total sample size was calculated to be 864 patients (432 patients per group). After considering the uncertainty in key parameter estimates and the feasibility and operational convenience of multicenter recruitment, we made a moderate adjustment to the sample size; the final plan is to enroll 900 patients (450 patients per group). Considering that wAMD and DME share comparable pathophysiological mechanisms (both involving the VEGF pathway) and primary efficacy end points (change in BCVA letter count), differences may exist between patient populations. We therefore conducted a sensitivity analysis based on published DME studies [21-27], adhering to the aforementioned criteria (a 2-sided test, with 1 – β = .9 and *α*=.05; the missed visit rate was set at 20%). The total sample size we calculated ranges from 58 to 886 patients, demonstrating that the selected parameters in our study, including effect size and variance, were appropriate. To ensure data comparability, hospitals were matched based on the number of annual ophthalmology outpatient visits. Patients were selected according to previously outlined inclusion and exclusion criteria. The primary outcome for sample size calculation was the mean change in BCVA from baseline to 12 months between the intervention and control groups.

### Management Description

#### Remote Management Description

**Figure 2.**
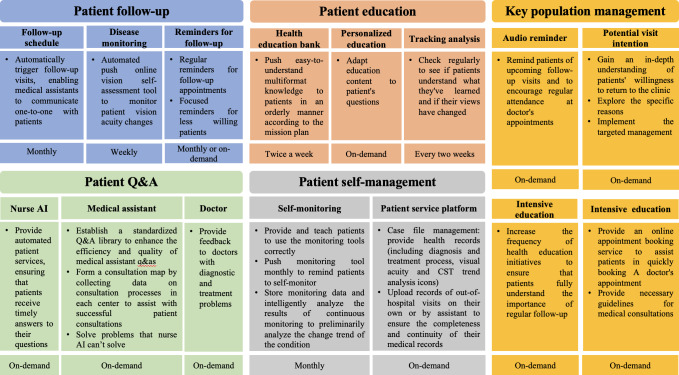
Structure of the patient remote management program and definition of key monitoring populations. AI: artificial intelligence; CRT: central retinal thickness; Q&A: questions and answers.

The patient remote management program comprised five primary domains: patient follow-up, patient education, patient Q&A, patient self-management, and key population management (Figure 2).

The remote management program categorized patients according to follow-up adherence during different treatment phases of intravitreal anti-VEGF therapy. The loading period was defined as the first 3 months following the initial intravitreal injection. Patients were classified as at risk of poor adherence when the interval between scheduled return visits exceeded 6 weeks. The maintenance period was defined as the period beginning 3 months after the first injection. Patients were considered at risk when the interval between follow-up visits exceeded 12 weeks. Patients were also classified into the risk group if they failed to provide feedback through the digital platform or did not respond after reminder notifications from medical assistants, even when the visit interval had not exceeded the predefined threshold. The remote management system incorporated monitoring, reminder, feedback, and physician-patient communication modules to support adherence and long-term treatment persistence.

#### Routine Management Description

Routine management measures primarily consisted of hospital medical professionals providing patients with disease education, either verbally or in writing, and informing them of their subsequent follow-up appointment time during each outpatient visit. This management was conducted mainly offline, meaning in-person and within the hospital setting. Patients did not routinely receive online disease education or telephone follow-up.

### Data Collection and Management

#### Data Collection

Table 1 presents the parameters and assessment times for the study in the remote management group.

Table 2 presents the parameters and assessment times for the study in the routine management group.

**Table 1.** The parameters and times of assessment for the study in the remote management group.

Parameters	Baseline	Fourth week	Eighth week	12th week	24th week	48th week
Sociodemographic information	✓					
Primary outcomes						
Best-corrected visual acuity	✓	✓	✓	✓	✓	✓
Secondary outcomes						
Central retinal thickness	✓	✓	✓	✓	✓	✓
Number of intravitreal injections		✓	✓	✓	✓	✓
Number of follow-up visits		✓	✓	✓	✓	✓
Compliance rate for follow-up visit		✓	✓	✓	✓	✓
Loss to follow-up rate		✓	✓	✓	✓	✓
Incidence of adverse events		✓	✓	✓	✓	✓

**Table 2.** The parameters and times of assessment for the study in the routine management group.

Parameters	Baseline	As prescribed	24th week	48th week
Sociodemographic information	✓			
Primary outcomes				
Best-corrected visual acuity	✓	✓	✓	✓
Secondary outcomes				
Central retinal thickness	✓	✓	✓	✓
Number of intravitreal injections		✓	✓	✓
Number of follow-up visits		✓	✓	✓
Compliance rate for follow-up visit		✓	✓	✓
Loss to follow-up rate		✓	✓	✓
Incidence of adverse events		✓	✓	✓

#### Sociodemographic Information

The researchers devised a questionnaire to collect the sociodemographic data from the participants. This study collected sociodemographic data from the participants, including their age, marital status, health status, and other relevant demographic information. The questionnaire included a column where participants were asked to list any medicines or supplements they were currently taking, as well as any symptoms or illnesses they were experiencing.

#### Primary Outcome

The primary outcome was the BCVA as measured using the ETDRS test, which was used to compare the change in visual acuity at each time point before and after medication.

#### Secondary Outcomes

The secondary outcomes included the following:

- CRT was measured using optical coherence tomography.
- The number of intravitreal injections was determined by calculating the number of intravitreal anti-VEGF injections administered to patients from enrollment to the end of the follow-up cycle (12 mo), as advised by medical professionals.
- The number of follow-up visits was determined by calculating the number of visits from enrollment to the end of the follow-up cycle (12 mo), as advised by medical professionals.
- The compliance rate for follow-up visits was the proportion of patients whose each follow-up visit was no more than 2 weeks from the scheduled follow-up date, calculated during the observation period after the patient initiates treatment, as advised by medical professionals.
- The loss to follow-up rate was calculated based on the number of patients who did not visit the hospital during the observation period following treatment initiation. This was defined as a period of more than 2 months without a follow-up visit to the hospital during the observation period, until the end of the observation period.
- The incidence of adverse events was defined as the proportion of adverse medical events in a study patient that were not necessarily causally related to the intravitreal injection, such as death or life-threatening events, or those resulting in persistent or significant loss of function.

#### Data Management

The data was carefully stored in a password-protected, secure electronic research database, which was supported by a backup system, antivirus software, and security protocols to help ensure data safety. Access to the electronic database was thoughtfully limited to a select group of researchers, who were listed in the authorization log of the survey master file. The data manager kindly oversaw regular data quality control checks and generated reports and statistics using tools integrated within the survey distribution platform.

### Statistical Analysis

#### Analyses of Outcomes

The collected data were entered into Excel 16.0 (Microsoft Corporation), and SPSS 25.0 (IBM Corp) was subsequently used for data analysis. The measurement data were presented as means and SDs, and the count data were expressed as percentages. Before primary analysis, continuous variables (eg, visual acuity change values) underwent normality testing using a combination of the Shapiro-Wilk test and Q-Q plots. Should the data be found to be normally distributed, parametric tests will be applied. For data showing significant deviation from normality, appropriate transformations or nonparametric tests were considered. To account for the multicenter design and potential clustering effects across different cities and hospitals, linear mixed-effects models were used, with the treatment group as a fixed effect and the center/hospital as a random effect. Statistical significance was considered as a *P* value less than .05. When reporting intergroup comparisons, *P* values were accompanied by corresponding effect sizes (eg, Cohen *d*) and their 95% CIs to assess the clinical relevance of observed differences.

#### Handling Missing Data

The missing data were addressed with multiple imputation methods, assuming random data loss. Sensitivity analyses were conducted to assess the robustness of the results, and a full case analysis was performed for comparison. Furthermore, where applicable, data missing due to patient dropout were reported, and any deviations from the original analysis plan were thoroughly documented.

## Results

This study integrates the BCW framework with the technical characteristics of digital platforms to systematically design and evaluate interventions. The modeling process comprises three core steps: (1) conducting a behavioral diagnosis and analysis based on the BCW, (2) mapping theory to digital functional design, and (3) developing a digital remote management platform. The platform integrates multiple functional modules, including patient education, treatment reminders, follow-up monitoring, symptom reporting, and doctor-patient communication. These have been designed to support long-term adherence and promote continuous disease management. This system architecture facilitates real-time interaction between patients and health care providers, delivering personalized feedback and remote monitoring throughout the treatment period. A total of 1006 patients with DME underwent initial screening, and 958 (95.2%) patients met the inclusion criteria, with only 7 patients declining follow-up. Participant recruitment began in September 2023 and was completed by the time of submission. The data analysis is yet to begin. The results are expected to be published in 2026.

Because this is a study protocol describing an ongoing intervention, the primary focus of the initial analysis will be on the feasibility of the remote management system, patient engagement with the smartphone app, and the acceptability of the BCW-based intervention content. We have established several benchmarks to define the success of this management model: we anticipate an enrollment rate of over 60% among eligible patients and a retention rate of at least 80% for the 12-month follow-up period. Regarding intervention adherence, we expect that at least 75% of participants will actively use the self-monitoring features (such as the digital Amsler grid) at least once a week and that the majority (>70%) will interact with the personalized health education modules.

We hypothesize that patients enrolled in the remote management program will demonstrate significantly higher treatment adherence, characterized by a lower rate of missed intravitreal injection appointments and more frequent follow-up visits, compared to a historical control group. We also expect that the intervention group will achieve superior visual outcomes, specifically a greater mean improvement in BCVA and a more significant reduction in CRT at the 12-month time point. Furthermore, we will explore the impact of the intervention on patient-reported outcomes, including self-efficacy in managing diabetes and vision-related quality of life. Although this real-world study primarily evaluates clinical effectiveness, we will examine the effect sizes of the remote management model to determine its potential for large-scale clinical implementation.

## Discussion

We hypothesize that the RE-SHINE remote management model will significantly enhance treatment adherence and improve clinical outcomes in patients with DME. Specifically, by addressing the barriers identified through the BCW framework, we anticipate a measurable increase in follow-up completion rates and a reduction in missed anti-VEGF injection appointments. These improvements are expected to translate into better long-term preservation of BCVA and a more consistent reduction in CRT compared to patients receiving routine clinical care. Of note, the efficacy of remote management has been demonstrated in several areas, including facilitating improved patient adherence to treatment [28], enhancing disease prognosis [29,30], managing health issues and data, providing personalized self-care advice, supporting patient-provider communication, and decision-making [31]. Despite these promising results, there is a paucity of clinical studies on this subject in patients with DME who received intravitreal anti-VEGF therapy. Building on the team’s previous in-depth study, this study was designed to evaluate the utility and effectiveness of management measures using a cohort study.

Interestingly, our study is specifically tailored for the intensive treatment phase of DME. Building on our previous qualitative research that identified key barriers such as injection anxiety and travel burden [4], the RE-SHINE program integrates targeted psychological support and real-time vision monitoring. This transition from “theory-based barrier identification” to “evidence-based digital intervention” represents a significant advancement over generalized teleophthalmology models.

By addressing the “psychological capability” through education, “social opportunity” through patient Q&A, and “physical opportunity” via remote monitoring, the RE-SHINE program directly targets the barriers identified in our framework, aiming to translate theoretical determinants into improved clinical adherence.

The management methods used in this study included a remote virtual approach; a patient relationship management tool to facilitate task shifting; and a scaled, team-based management approach that incorporated physician assistants, nurse practitioners, and physicians who oversaw and intervened in management as required, in addition to self-monitoring of the patient’s condition. Given that patients undergoing anti-VEGF therapy spend the majority of their time outside of the hospital settings, remote management can meet their needs when needed.

Self-monitoring tools enable patients to acquire specialized health knowledge, track the empowering effects of their health indicators, and improve their ability to access resources [32,33]. This study hypothesizes that self-monitoring will help patients track their disease progression before and after intravitreal injections, improve the efficacy of remote management, and support patients in overcoming challenges during treatment.

The patient’s adherence to treatment has been demonstrated to have a significant influence on disease outcomes [29,30]. This study involves remote management at various points throughout the treatment period, with a focus on the changes that occur during the entire therapy cycle.

This study is one of the first systematic attempts to integrate a BCW-based remote management model into real-world anti-VEGF therapy for patients with DME in China. It has the potential to establish a foundational framework and provide a series of concepts that will inform subsequent studies on the management of treatment adherence to anti-VEGF in patients with DME. The findings of this study have the potential to directly influence patients’ disease outcomes by enhancing their adherence to treatment regimens. The potential economic benefits of increased adherence may be reflected in a reduced demand for health care services, resulting in lower expenditures.

A major strength of this study is its solid theoretical foundation using the BCW, which ensures that the digital intervention directly targets the modifiable behaviors of patients with DME. Additionally, the multicenter design involving 26 cities enhances the real-world generalizability of our findings. However, several limitations must be acknowledged. First, the sample size estimation for this study was based on hypothetical parameters derived from the wAMD population, which may be subject to inherent limitations. These include potential differences in patient demographics (such as age distribution), real-world treatment patterns and intervals, and treatment adherence or discontinuation behaviors. Such variations may have a significant impact on the estimated effect size and outcome variability. Future studies should focus on obtaining more precise parameter estimates within the DME population. Second, the effect of remote management is only tracked in the first year of treatment, so the validity of its long-term maintenance still needs to be further validated. Third, the study may be limited due to the “digital divide,” and patients with low digital literacy or those without access to smartphones may face barriers to participation. Additionally, while the platform’s features are evidence-based, a formal standardized usability and acceptability assessment of the app interface was not the primary objective of this protocol phase.

## Supplementary material

10.2196/85764 Checklist 1 SPIRIT checklist.

## Abbreviations

BCVA: best-corrected visual acuity
BCW: Behavior Change Wheel
COM-B: capability, opportunity, motivation–behavior
CRT: central retinal thickness
DME: diabetic macular edema
ETDRS: Early Treatment Diabetic Retinopathy Study
Q&A: questions and answers
VEGF: vascular endothelial growth factor
wAMD: wet age-related macular degeneration
